# Revisiting HPV integration landscapes using telomere-to-telomere human genome reference closes the gaps in HPV integration sites search

**DOI:** 10.1186/s12985-026-03241-y

**Published:** 2026-07-09

**Authors:** Alicja Pacholewska, Bushra Yusuf Khan, Michal R. Schweiger

**Affiliations:** 1 Institute for Translational Epigenetics, Faculty of Medicine and University Hospital Cologne, University of Cologne, Weyertal 115B, Cologne, 50931 Germany; 2 Center for Molecular Medicine Cologne (CMMC), Medical Faculty, University Hospital Cologne, University of Cologne, Robert-Koch-Str. 21, Cologne, 50931 Germany; 3 Cologne Center for Genomics (CCG) and West German Genome Center (WGGC), University of Cologne, Weyertal 115B, Cologne, 50931 Germany

**Keywords:** HPV, Integration, Breakpoint, Hs1, Centromeres, Satellites, Repeats

## Abstract

Accurate characterization of human papillomavirus (HPV) integration into the host genome is essential for understanding HPV-driven carcinogenesis. However, most previous integration studies relied on incomplete human reference genomes, potentially obscuring integration events occurring within highly repetitive genomic regions. Here, we reassessed HPV integration landscapes using the first complete telomere-to-telomere (T2T) human reference genome assembly (hs1). We reanalyzed datasets from three independent studies generated using complementary sequencing technologies, including short-read Illumina sequencing, PacBio continuous long reads (CLR), PacBio high-fidelity (HiFi) sequencing, and ultra-long Oxford Nanopore sequencing. Reanalysis using the T2T reference confirmed the majority of previously reported integration breakpoints while identifying numerous additional sites, increasing the total number of detected breakpoints from 446 to 799. Notably, newly detected integrations frequently localized to repeat-rich genomic regions, including centromeric and pericentromeric satellite sequences that were absent from earlier reference assemblies. Importantly, integration events within these regions were detectable not only in long-read datasets but also in short-read sequencing data when analyzed using the complete reference genome. Our findings demonstrate that incomplete reference assemblies have led to systematic underestimation of HPV integration events, particularly within structurally complex genomic regions. These results highlight the importance of adopting complete human genome references for viral integration analyses and suggest that reanalysis of existing datasets may reveal previously overlooked biologically and clinically relevant integration events.

**Supplementary Information:**

The online version contains supplementary material available at https://doi.org/10.1186/s12985-026-03241-y.

## Introduction

Persistent infection with high-risk human papillomaviruses (HPVs), particularly HPV16 and HPV18, represents a major driver of human carcinogenesis and is causally linked to cervical cancer as well as a substantial fraction of anogenital and oropharyngeal malignancies [1, 2]. A critical event associated with malignant progression is the integration of viral DNA into the host genome, which can disrupt genomic integrity, alter host gene regulation, and promote sustained expression of the viral oncogenes E6 and E7 [3]. Consequently, accurate identification of HPV integration sites is essential for understanding mechanisms underlying HPV-driven tumor development.

HPV integration is not part of the productive viral life cycle and is thought to occur as a consequence of host DNA repair processes following double-strand breaks [4]. Proposed mechanisms include non-homologous end joining and microhomology-mediated end joining, facilitated by microhomologies between viral and host DNA [5]. A looping model suggests that viral-host DNA forms unstable loop-like intermediates that can undergo amplification, excision, and reintegration, generating complex viral-host concatemers [6]. More recent work described “heterocatany,” in which viral-host concatemers exist as extrachromosomal circular DNA (ecDNA), recombine with other ecDNAs, and reintegrate into the genome, contributing to genomic instability [7]. Previous studies have demonstrated preferential integration near transcriptionally active loci, common fragile sites, and regions prone to replication stress [3]. However, the full genomic landscape of HPV integration remains incompletely resolved. Integration frequencies differ between cancers, occurring in ~ 80% of cervical carcinomas but only ~ 13% of HPV-positive head and neck squamous cell carcinomas (HNSCC) [8], although recent study has shown integration in over 50% of HPV-positive HNSCC patients’ samples [9]. Recurrent hotspots include genes such as *MYC*, *RAD51B*, and *TP63* [10]. Integration may result in transcriptionally active viral-host fusion transcripts, often associated with upregulation of nearby host genes [11]. Epigenetic modifications, such as methylation of E2 binding sites, can also enhance E6 and E7 expression even without integration [12].

A major limitation of earlier integration studies lies in the incompleteness of previous human reference genome assemblies. Nearly half of the human genome consists of repetitive DNA, including satellite repeats located within centromeric and pericentromeric regions [13]. These sequences were largely absent or poorly assembled in commonly used references such as hg19 and hg38, preventing reliable mapping of sequencing reads originating from these regions. As a result, viral integration events occurring within repeat-rich genomic contexts may have remained undetected.

Recent advances in genome assembly culminated in the telomere-to-telomere (T2T) human reference genome (chm13v2/hs1), which provides the first gapless representation of all human chromosomes, including previously unresolved centromeric and satellite sequences [14]. This resource enables systematic investigation of genomic regions that were inaccessible to earlier analyses and offers an opportunity to reassess viral integration landscapes using existing sequencing datasets.

In parallel, long-read sequencing technologies have substantially improved the resolution of complex structural variants and viral-host junctions [7, 15]. While short-read sequencing has enabled large-scale detection of HPV integrations, it remains limited in resolving repetitive regions and structurally complex rearrangements. Long-read approaches, including PacBio and Oxford Nanopore sequencing, overcome many of these limitations and allow more accurate reconstruction of integration events. Nevertheless, even long-read studies performed prior to the availability of the T2T reference genome were constrained by incomplete genomic references.

In this study, we evaluated the impact of the complete T2T human reference genome on HPV integration detection by reanalyzing datasets from three independent studies generated using various sequencing strategies: short-read Illumina sequencing, PacBio continuous long reads (CLR), PacBio HiFi sequencing supported by Oxford Nanopore ultra-long reads. By remapping these datasets to the hs1 reference genome, we aimed to determine to what extent HPV integration events were previously missed due to limitations of earlier genome assemblies and to assess whether repeat-rich genomic regions represent an underappreciated component of the HPV integration landscape.

## Results

### Overview of analyzed datasets and sequencing strategies

To evaluate the impact of reference genome completeness on HPV integration detection, we reanalyzed previously published datasets representing diverse experimental designs and sequencing technologies. The analyzed studies comprised HPV-positive cervical cancer and oropharyngeal squamous cell carcinoma samples generated using both targeted and untargeted sequencing approaches.

The dataset from Iden et al. [16]. employed HPV-enriched PacBio continuous long-read (CLR) sequencing to characterize integration events in cervical cancer samples, enabling detection of extended viral–host junctions despite relatively lower per-read accuracy. In contrast, Akagi et al. [7]. analyzed oropharyngeal tumor samples and four cell lines (HeLa, VU147, GUMC-395, and HTEC) using whole-genome long-read sequencing, including PacBio high-fidelity (HiFi) and Oxford Nanopore Technologies (ONT) sequencing, primarily to investigate structural rearrangements and clonal evolution associated with HPV integration. Finally, Demers et al. [17]. applied a proximity ligation–based short-read Illumina sequencing approach optimized for formalin-fixed paraffin-embedded (FFPE) oropharyngeal squamous cell carcinomas material to identify viral integration sites in clinical specimens.

Together, these datasets encompass four complementary sequencing strategies differing in read length, enrichment strategy, and sequencing accuracy. This diversity enabled systematic assessment of whether previously undetected HPV integration events arise primarily from technological limitations or from incomplete representation of the human reference genome. All datasets were therefore uniformly remapped to a hybrid reference consisting of the telomere-to-telomere human genome assembly (chm13v2/hs1) and the HPV16 reference genome prior to downstream breakpoint analysis.

Having established a harmonized analytical framework across sequencing platforms and experimental designs, we next assessed how the use of the complete telomere-to-telomere reference genome affects the detection and annotation of HPV integration events compared with the original hg19-based analyses.

### Reanalysis using the T2T reference genome reveals additional HPV integration breakpoints

**Fig. 1:**
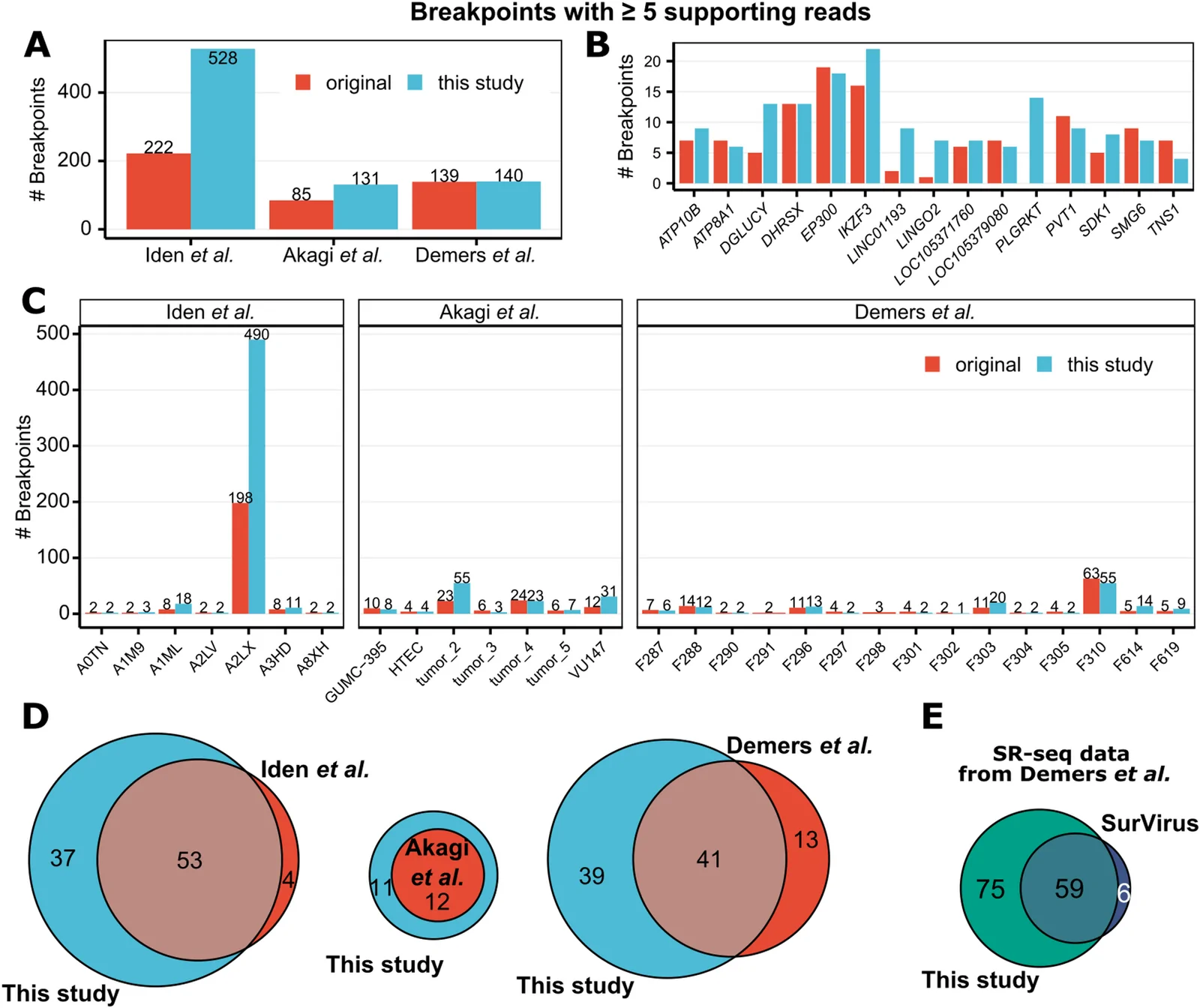
Breakpoints identified in this and original studies. **A**. The number of all breakpoints identified in each study with at least 5 supporting reads (when applicable). **B**. The number of identified breakpoints from A shown for unique ten most frequent genes overlapping the breakpoints identified in original (red) and this study (blue). **C**. The number of identified breakpoints from A shown for each sample separately. **D**. The overlap between unique chromosomal cytobands originally reported with identified breakpoints. For each study a venn diagram shows the number of cytobands with breakpoints with at least 5 supporting reads identified in this study (red, hg19) and original study (blue, hs1). **E**. The overlap between breakpoints with at least 5 supporting reads identified in this project by own pipeline (green) and by using SurVirus [24] (blue) for the short-read sequencing data from Demers et al. using the hs1 reference

Across all datasets, remapping to the T2T reference resulted in an increased number of detected viral–host breakpoints compared with the original analyses. In total, 799 integration breakpoints were identified, compared with 446 breakpoints reported in the original studies (Fig. 1A).

Despite the overall increase in breakpoint detection, the overlap between genes affected by HPV integration in the original and reanalyzed datasets remained high (Supplementary Figure S1). Among the ten genes most frequently associated with integration events, six were shared between analyses, including *DGLUCY*, *DHRSX*, *ATP10B*, *LINC01193*, *PVT1*, *LINGO2*, *EP300*, and *IKZF3*, with *EP300* and *IKZF3* representing the most frequently affected loci in both analyses (Fig. 1B). Comparison with entries in the Viral Integration Site DataBase, VISDB [18], a manually curated database of viral integration sites in the human genome, further confirmed that integrations within several of these genes have previously been reported, supporting the biological validity of the newly detected breakpoints: *DGLUCY* (*n* = 4, RNA-seq-based, head and neck squamous cell carcinoma samples) [19], *ATP10B* (*n* = 1, high-throughput viral integration detection (HIVID)-based [20]) [21], *PVT1* (*n* = 5, cervical cancer samples, PCR & Sanger sequencing-based) [22], *LINGO2* (*n* = 3, HIVID-based [20]) [21]; and *IKZF3* (*n* = 1, squamous cell carcinoma samples, RNA-seq & RT-PCR-based) [23]. The general overlap between genes for which breakpoints were identified in this and original studies was very good (Supplementary Figure S1), although there were just single genes that appeared in more than one of the three studies (Supplementary Table S1), suggesting the genes were more patient-specific rather than indicating a true hotspot gene.

Although additional breakpoints were detected overall, increases were not uniformly distributed across all samples. In 11 of 29 analyzed samples, more breakpoints were identified than originally reported, with the largest contribution arising from a single sample in the Iden et al. dataset (Fig. 1C). Importantly, 32 newly detected breakpoints could not be lifted over to the hg19 reference genome, indicating that these integration events occur within genomic regions absent from earlier assemblies and therefore could not have been detected in the original analyses.

It is known that HPV integrations may differ in numbers among affected individuals. It can be caused by prolong HPV infection, high viral loads, host genomic instability and DNA damage, or other environmental factors. Even after exclusion of the A2LX sample, in which both original and this study identified outlying high number of integrations, the number of integration sites is slightly higher in our compared to original studies (Fig. 1C). Nevertheless, we had a closer look at the additional breakpoints identified in samples with > 1.5-fold increase compared to original studies.

In the A2LX sample, we identified 75 integrations within cenSat regions (from which 23 were missing in hg19) and only 13 were identified in the original study. For samples tumor_2 and VU147 most of the additional integrations (12 out of 36) were observed based on ONT reads. Whereas in sample VU147 we identified 19 integrations, the original study reported only 6 integrations based on ONT reads. Three additional integrations on chr15 were identified based on both ONT and PacBio HiFi reads, whereas they were not reported in the original study. In tumor_2 sample we identified 12 additional integrations based on ONT reads on chr9 p24.1, mostly near the *PLGRKT* gene (Supplementary Table S1).

For the F614 sample, we could not find any trend of the additional 9 breakpoints identified – only one of these could not be mapped to hg19 reference and three were identified on chrX – in the original study there were no breakpoints identified on chrX, although there were four breakpoints identified on chrY in two samples, three of which were confirmed by our analysis (Supplementary Table S1).

Because breakpoint coordinates depend on the reference genome used for alignment, direct positional comparison between hg19- and hs1-based analyses is challenging. To enable systematic comparison, breakpoints occurring within the same cytogenetic band in both assemblies were considered to represent potentially identical integration events. Using this approach, we recovered the majority of previously reported integration sites (Iden et al., 93%; Akagi et al., 100%; Demers et al., 76%) while identifying additional cytogenetic regions harboring HPV integrations (Fig. 1D). Notably, 11% of originally reported breakpoints did not match the expected cytoband after lift-over to hs1, highlighting the impact of reference genome choice on integration site annotation. All the breakpoints with cytoband annotations per samples are shown in Supplementary Figures S2-S4. Although we tried to follow the original thresholds for calling a breakpoint, the details were not always revealed. In our analysis we kept breakpoints supported by at least 5 reads. All breakpoints identified are listed in Supplementary Table S1.

For short-read sequencing datasets, we additionally compared our results with integration calls generated using SurVirus [24], a repeat-aware integration detection algorithm optimized for short-read data. Our analysis recovered approximately 90% of SurVirus-identified breakpoints (Fig. 1E). However, the lack of realignment step in our strategy resulted in the identification of 75 additional breakpoints, supported by an average of 15 reads (Supplementary Table S1), suggesting that integration detection sensitivity may increase when complete reference genomes are combined with permissive breakpoint-support thresholds. When we applied more stringent threshold of minimum 10 supporting reads, our method identified 33 more breakpoints, whereas SurVirus identified three more. When we increased the threshold to 20, the numbers decreased to 10 and 1, respectively.

The six breakpoints identified solely by the SurVirus using the initial threshold of minimum five supporting reads were located in repeat-rich regions: three within cenSat annotated regions, and the other three within repetitive regions annotated by repeatMasker (version April 01 2021 open-4.1.2-p1) [25]. These breakpoints were likely missed in our analysis due to the read sparsity within repeats leading to low read coverage.

Collectively, these results demonstrate that reanalysis of existing sequencing datasets using the complete T2T human genome assembly enables recovery of previously undetected HPV integration events. Importantly, additional breakpoints were identified not only in long-read datasets but also in short-read sequencing data, indicating that incomplete reference assemblies rather than sequencing technology alone have contributed to underestimation of HPV integration landscapes.

### Breakpoints in repeat-rich and satellite regions are revealed by the T2T reference genome

Previous human reference genome assemblies lacked large portions of repeat-rich genomic regions, including centromeric and pericentromeric satellite sequences. Because these regions are inherently difficult to assemble and map, HPV integration events occurring within them have likely been systematically underestimated. We therefore examined whether the additional breakpoints detected using the T2T reference genome were enriched within satellite repeat regions.

**Fig. 2:**
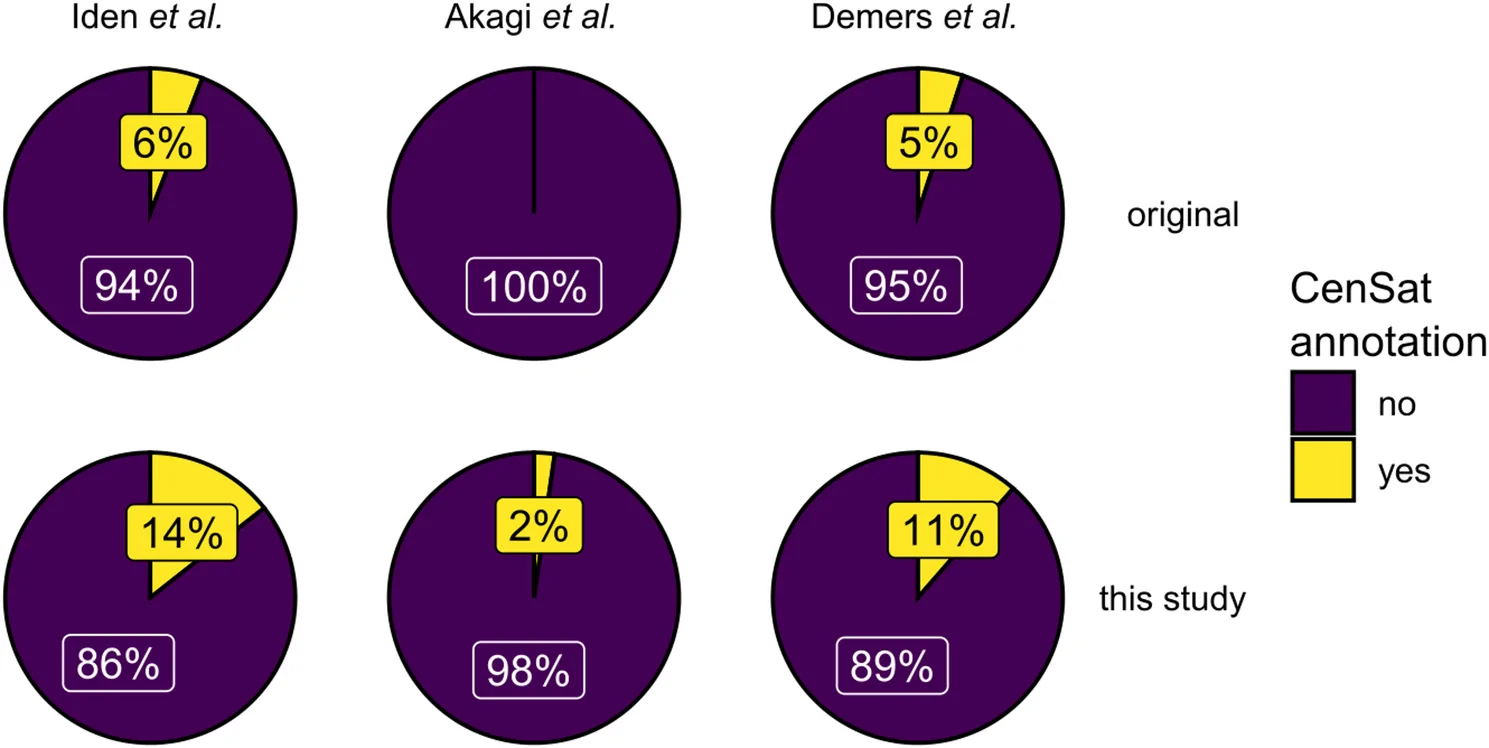
Proportion of breakpoints identified in satellite (cenSat) regions. For each study a pie chart shows the percentage of breakpoints (at least 5 supporting reads) within and outside cenSat annotation is provided based on original studies (top) and this study (bottom)

Across all datasets, we identified a total of 95 HPV integration breakpoints located within centromeric satellite (cenSat) annotations (Fig. 2). Although a statistically significant increase in satellite-associated integrations compared with the original studies was observed primarily for the Iden et al. dataset, additional cenSat-associated breakpoints were detected in all reanalyzed datasets. Importantly, a substantial fraction of these breakpoints mapped to genomic regions that were absent from the hg19 reference genome, including 38% of cenSat breakpoints identified in the Iden et al. dataset and 30% in the Demers et al. dataset (Supplementary Table S1). These integration events therefore could not have been identified in earlier analyses.

The highest number of satellite-associated breakpoints was detected in the A2XL sample from the Iden et al. study, in which 75 cenSat integrations were identified. While the original study reported 13 satellite-associated breakpoints after lift-over to hs1 coordinates, our reanalysis additionally revealed integrations in other samples, including one breakpoint in sample A1ML supported by five independent reads (Supplementary Table S1).

**Fig. 3:**
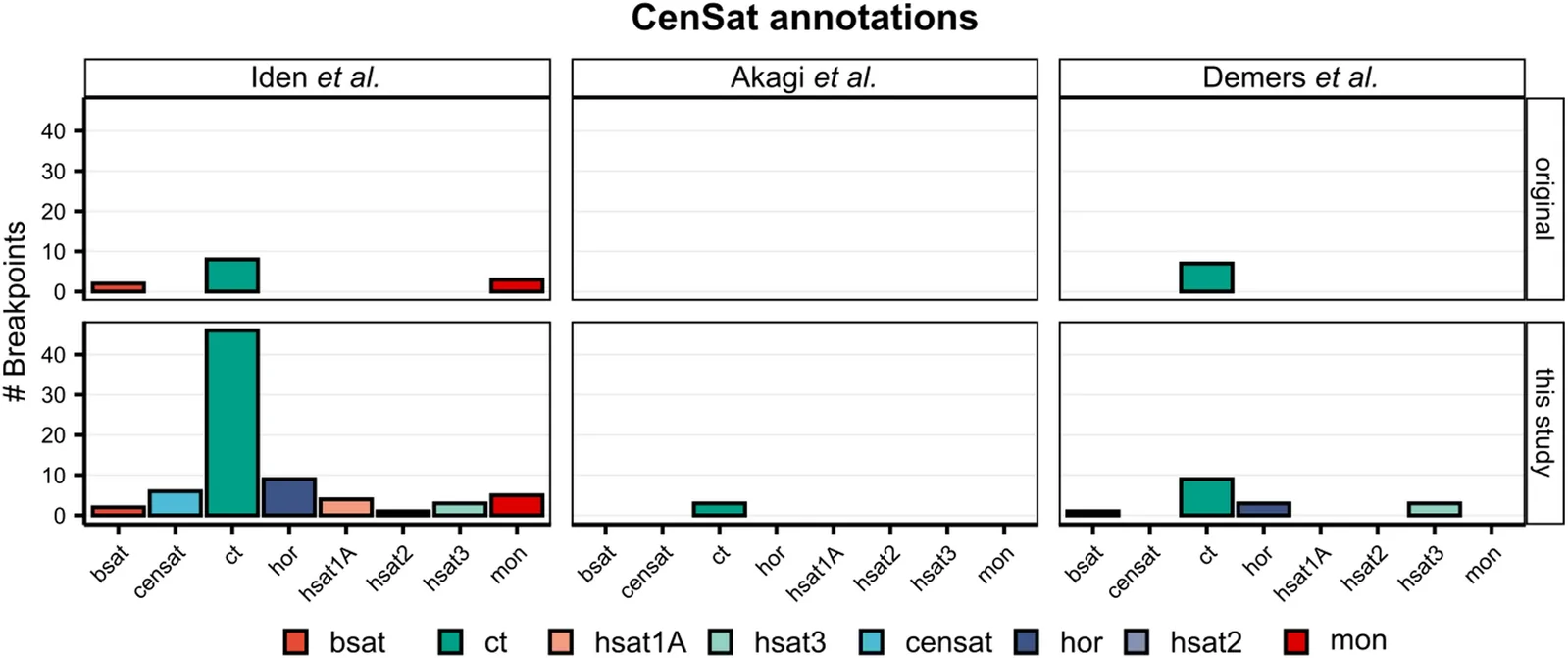
Breakpoints within satellite regions. Bar plots with breakpoints counts per satellite type separated by each study. Bsat – beta satellite; ct centromeric transition region; hsat1A – classical human satellite 1 A; hsat3 – classical human satellite 3; censat – other centromeric satellite; hor – alpha satellite higher order repeat; hsat2 – classical human satellite 2; mon – monomeric alpha satellite

**Fig. 4:**
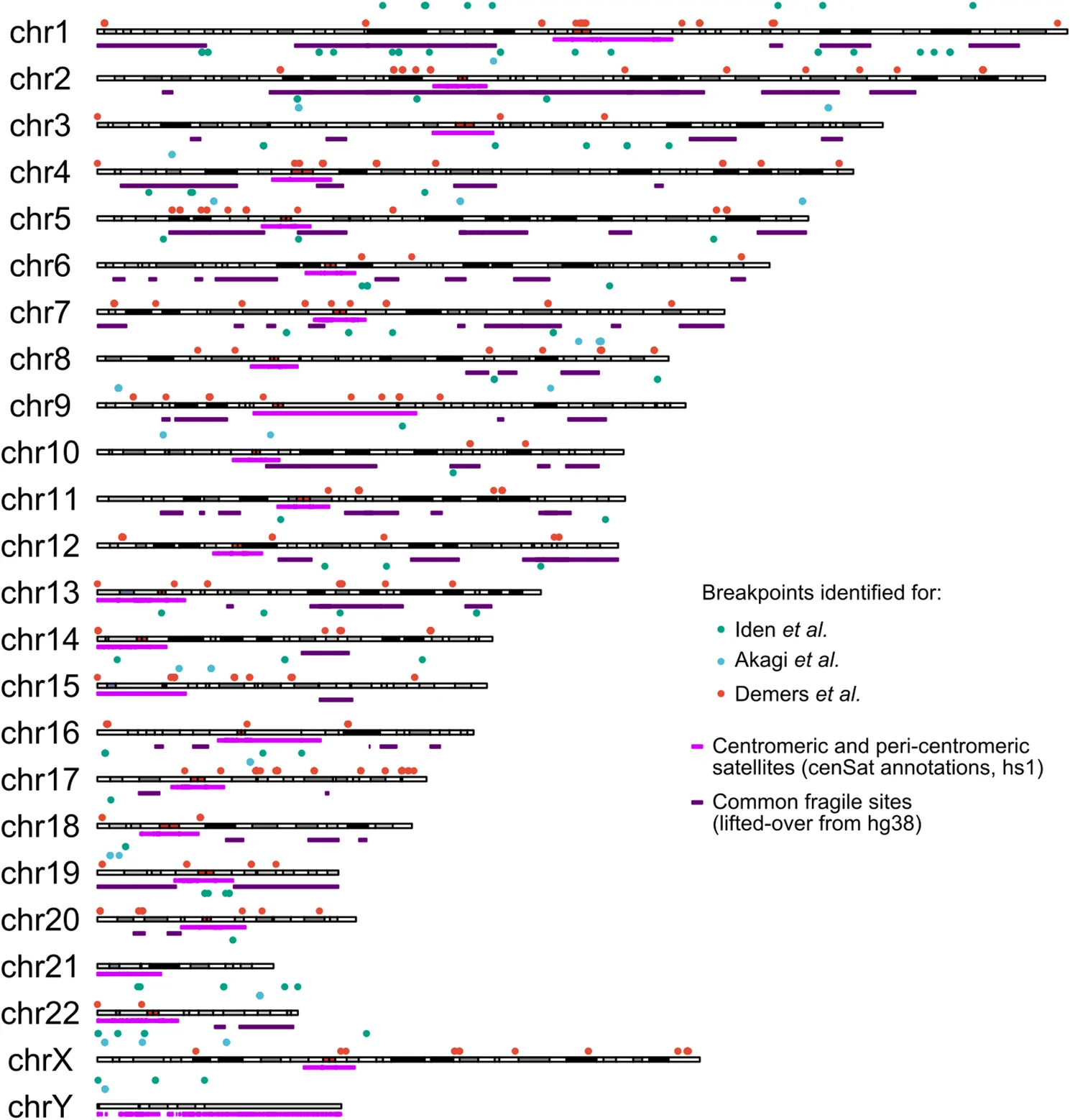
Karyogram indicating breakpoints identified in this study. The dots above each chromosome indicate the breakpoints from Iden et al. (red), Akagi et al. (blue), and Demers et al. (green). Bars below the chromosomes indicate cenSat annotations for hs1 (magenta) or common fragile sites annotated for hg38 and lifted-over to hs1 (purple)

Classification of satellite-associated integrations revealed that most breakpoints localized to centromeric transition regions (61%), followed by alpha satellite higher-order repeat arrays (13%) (Figs. 3 and 4). These regions are characterized by elevated replication stress and structural instability, features that have previously been linked to chromosomal rearrangements in cancer genomes [26].

Notably, analysis of unfiltered breakpoints from the Iden et al. dataset indicated that approximately 44% of detected integrations were located within repetitive regions, substantially exceeding the estimated genomic coverage of centromeric repeats (~ 14.5%). Statistical testing confirmed significant enrichment of integrations within these regions (one-sample proportion test (X² = 2,582.7, p-value < 2.2e-16)), suggesting that centromeric repeats may represent preferential targets of HPV integration. However, read support within repetitive regions was frequently reduced due to multimapping effects, preventing many candidate integrations from reaching stringent coverage thresholds.

Inspection of breakpoint distributions within repetitive regions revealed a recurring clustering pattern. Integration breakpoints showed highly consistent positions within the HPV genome, whereas the corresponding host genomic coordinates were dispersed along repeat arrays (Supplementary Figure S5). This pattern was largely restricted to repeat-containing regions and was typically supported by low read coverage, leading to exclusion of many events during filtering. Similar clustering patterns have been reported previously and may reflect post-integration genomic instability or looping interactions between viral and host DNA that generate locally amplified viral–host junctions [6].

Alternatively, this apparent dispersion of host breakpoints may arise from technical limitations in read alignment within repetitive DNA, where sequencing errors or natural sequence variation can result in ambiguous mapping positions. Nevertheless, the reproducible detection of viral breakpoint positions across independent reads supports the presence of genuine integration events within satellite regions.

Together, these findings demonstrate that the use of the complete T2T human reference genome substantially improves detection of HPV integrations within repeat-rich genomic regions. Approximately one-third of satellite-associated breakpoints identified here were inaccessible when earlier reference assemblies were used, highlighting the extent to which HPV integration landscapes have previously been underestimated regardless of sequencing read length.

### HPV integration breakpoints frequently occur within common fragile sites

Certain genomic regions are particularly susceptible to structural instability and DNA breakage. Common fragile sites (CFSs) represent chromosomal loci prone to replication stress and are frequently associated with genomic rearrangements in cancer genomes [3]. Because HPV integration has previously been linked to genomic instability [3, 27], we next investigated whether integration breakpoints identified in this study preferentially overlapped with annotated CFS regions.

**Fig. 5:**
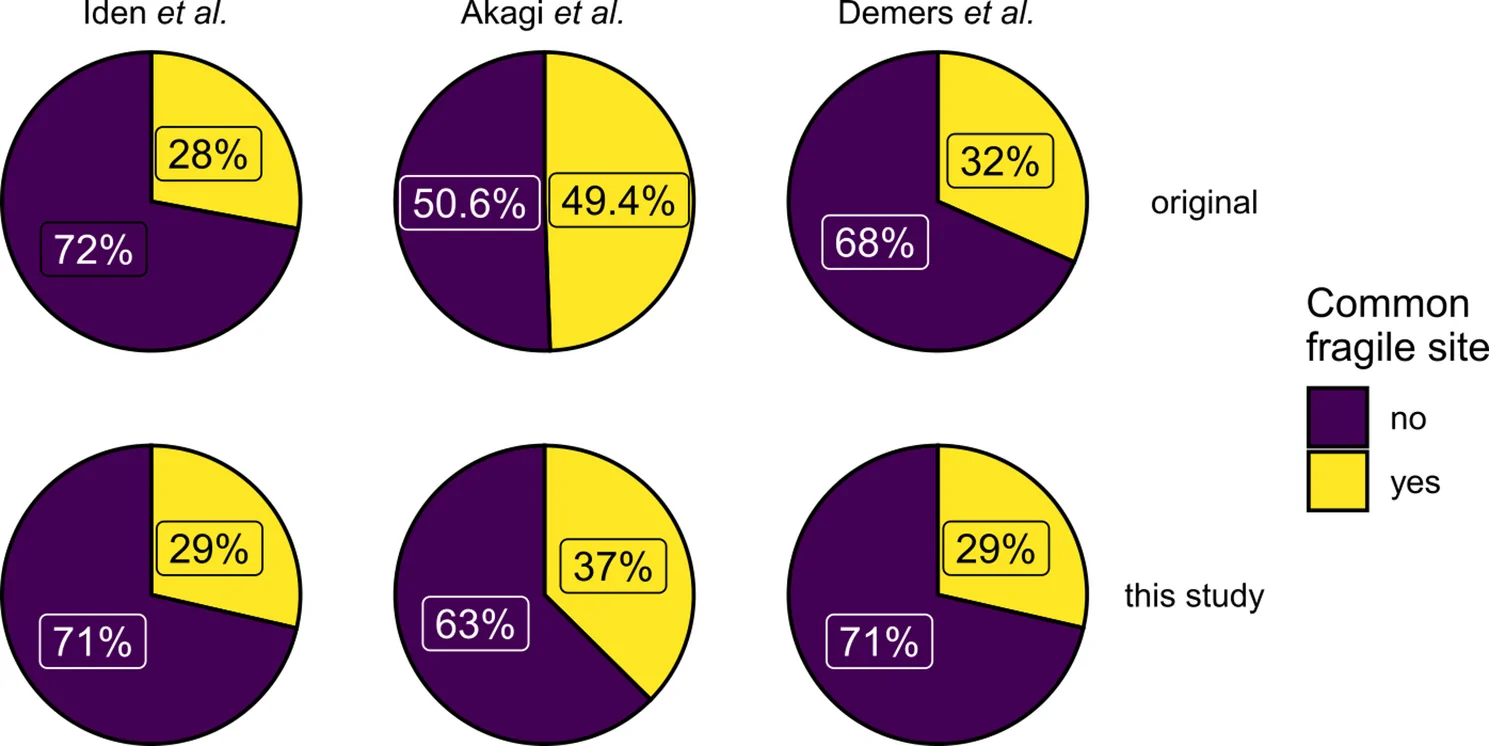
Proportion of breakpoints identified in common fragile sites (CFS). For each study a pie chart shows the percentage of breakpoints (at least 5 supporting reads) within and outside CFS annotation is provided based on original studies (top) and this study (bottom). CFS regions were lifted over from hg38 to hs1 reference

Comparison of breakpoint coordinates with known CFS annotations revealed that a considerable fraction of HPV integration events occurred within fragile genomic regions (Fig. 5). This trend was most pronounced in the Akagi et al. dataset, although the proportion of integrations overlapping CFSs was slightly lower than reported in the original analysis. Given that CFSs collectively span approximately 27% of the hs1 genome, statistical testing demonstrated significant enrichment of integration events within CFS regions only for the Akagi et al. dataset (2-sample test for equality of proportions with continuity correction X² = 7.4971, p-value = 0.00309, alternative hypothesis: greater).

Centromeric and pericentromeric repeat regions share several structural and replication-associated characteristics with CFSs, including increased replication stress and susceptibility to chromosomal rearrangements [26, 28]. We therefore examined the overlap between integrations occurring in satellite repeat (cenSat) regions and annotated CFS loci. Only 17 of 95 satellite-associated breakpoints were annotated within both cenSat and CFS regions, indicating that these genomic features largely represent distinct classes of unstable loci.

To evaluate whether integration events collectively preferentially occur within structurally vulnerable regions, we combined breakpoints located in either cenSat or CFS annotations. Despite the known genomic instability associated with both region types, no significant enrichment was observed across all datasets when analyzed jointly. These findings suggest that while HPV integration frequently occurs within fragile genomic environments, susceptibility to integration may depend on additional local genomic or cellular factors beyond large-scale chromosomal fragility.

Together, these results indicate that HPV integration events are recurrently associated with genomic regions characterized by replication stress and structural instability, including both common fragile sites and centromeric repeats. However, the absence of consistent enrichment across datasets highlights the heterogeneous nature of HPV integration and underscores the importance of comprehensive genome references for unbiased detection of integration landscapes.

## Discussion

Persistent infection with high-risk human papillomaviruses (HPVs), particularly HPV16 and HPV18, represents a central driver of carcinogenesis in cervical and oropharyngeal malignancies [29]. Integration of HPV DNA into the host genome constitutes a key molecular event associated with malignant progression, as it promotes genomic instability and deregulated expression of viral oncogenes. Accurate characterization of HPV integration landscapes is therefore essential for understanding both tumor evolution and virus–host interactions.

In this study, we reassessed HPV integration sites using the first complete telomere-to-telomere (T2T) human reference genome assembly (hs1) [14]. By reanalyzing datasets generated using multiple sequencing technologies, including short-read and long-read approaches, we demonstrate that previously incomplete reference genome assemblies have led to systematic underestimation of HPV integration events (Fig. 1), in agreement with previous study evaluating integrations of hepatitis B virus using hg38 and hs1 references [30]. Importantly, the majority of newly detected breakpoints localized to genomic regions that were absent or poorly resolved in earlier references.

Direct comparison of HPV integration sites identified using different human genome assemblies is challenging, as breakpoint coordinates are inherently dependent on the reference genome used for alignment. Differences between hg19 and hs1 can result in coordinate shifts, altered mapping in repetitive regions, and changes in local genome annotation, making direct comparison at single-base resolution unreliable. To address this issue, we compared integration events at the cytogenetic band level rather than relying on exact genomic coordinates. Integration sites located within the same cytogenetic band were considered potentially identical loci. This approach minimizes the impact of assembly-specific coordinate shifts while preserving biologically meaningful genomic localization. Using this approach, we were able to identify most of the previously reported integrations, especially in studies based on long-read sequencing (Fig. 1D).

Prior to the availability of the T2T assembly, approximately 8% of the human genome remained unresolved, largely corresponding to highly repetitive regions [14]. Our findings show that a proportion of HPV integrations occur within these previously inaccessible genomic contexts. Approximately one-third of satellite-associated integration events identified here could not be mapped to hg19 coordinates, demonstrating that earlier analyses were intrinsically unable to detect these events regardless of sequencing technology or analytical pipeline.

Within repeat-rich regions, we observed characteristic clustering patterns in which viral breakpoint positions remained highly consistent while host genomic coordinates appeared dispersed along repeat arrays. One biological interpretation is that an initial HPV integration event may trigger subsequent secondary DNA breakage or amplification processes, potentially consistent with previously proposed looping or concatemer formation models [6, 7]. Such mechanisms could contribute to localized genomic instability and structural rearrangements frequently observed in HPV-associated cancers.

An alternative, non-mutually exclusive explanation relates to technical challenges inherent to mapping reads within repetitive DNA. Ambiguous alignment caused by sequence similarity across repeat arrays can distribute reads across nearby genomic positions, resulting in apparently dispersed host breakpoints. Nevertheless, the reproducible detection of identical viral breakpoint positions across independent reads supports the presence of genuine integration events rather than mapping artefacts alone.

HPV integration has long been associated with genomic instability and preferential localization to structurally vulnerable genomic regions such as common fragile sites [3]. Consistent with previous reports, a considerable fraction of integrations overlapped fragile genomic environments. However, enrichment was not uniform across datasets, suggesting that HPV integration is influenced by multiple factors including chromatin accessibility, replication stress, local DNA repair activity, and tumor-specific evolutionary pressures. However, the clinical relevance of these ‘gap’ integrations remains a subject of active debate; while our DNA-level mapping is more comprehensive, recent evidence suggests that transcribed viral–host fusion transcripts are the primary determinants of poor prognosis [9]. It remains to be determined whether these newly identified structural disruptions in repetitive regions contribute to malignancy through long-range chromatin remodelling or if they represent non-transcribed passenger events.

An additional factor that may contribute to the observed integration patterns is the chromatin state of the host genome. Recent work has demonstrated that episomal HPV genomes preferentially associate with transcriptionally active and accessible chromatin, including super-enhancers and other regulatory regions characterized by open chromatin architecture [31]. These regions substantially overlap known HPV integration hotspots and sites prone to double-strand DNA breaks. One possible explanation is that HPV genomes initially tether to accessible chromatin to maintain active viral transcription during persistent infection and subsequently become integrated at or near these sites when DNA damage occurs. Although highly speculative, it is conceivable that following integration, some loci may retain features of an open chromatin environment, potentially facilitating continued viral oncogene expression or influencing local chromatin organization. Further studies integrating chromatin accessibility, epigenetic profiling, and HPV integration mapping will be required to determine whether the chromatin landscape actively shapes integration site selection and the biological consequences of integration.

An intriguing observation of our study is the unexpectedly high number of HPV integrations detected within centromeric and pericentromeric satellite regions. Although these regions are traditionally considered transcriptionally inactive and enriched for heterochromatic histone modifications, including H3K9me3 and H4K20me3, increasing evidence suggests that centromeric domains are highly dynamic and frequently altered in cancer cells [32]. Epigenetic disruption of centromeric chromatin has been associated with replication stress, chromosome mis-segregation, micronucleus formation, and structural rearrangements, all of which can promote genomic instability [33, 34].

While HPV integrations have historically been linked to transcriptionally active genomic regions, super-enhancers, and accessible chromatin environments [10], our findings suggest that repetitive heterochromatic regions may represent an additional and previously underappreciated class of integration targets. One possible explanation is that integration is driven less by steady-state chromatin accessibility and more by local DNA damage and repair activity. Centromeric and pericentromeric regions are known to experience elevated replication stress and DNA repair events, particularly in cancer cells, creating substrates for non-homologous end joining and microhomology-mediated end joining pathways that have been implicated in HPV integration [5].

The biological consequences of integrations within these regions remain largely unknown. Even in the absence of direct disruption of protein-coding genes, HPV integration into satellite DNA could potentially affect higher-order chromatin organization, centromere integrity, or chromosome segregation fidelity. Such mechanisms may be particularly relevant given that chromosomal instability is a hallmark of HPV-associated cancers and has been linked to viral integration events and HPV-induced genome remodeling [3, 7]. Furthermore, centromeric and pericentromeric domains participate in long-range chromatin interactions and nuclear organization, raising the possibility that integrations in these regions could exert regulatory effects on distant genomic loci [34, 35].

Another possibility is that at least a subset of these integrations reflects ongoing genomic instability rather than classical driver events. However, the recurrent detection of integrations within centromeric transition regions across independent datasets and sequencing platforms argues against purely technical artefacts and suggests a biological basis for their occurrence. Centromeric transition regions are increasingly recognized as evolutionarily dynamic domains located between homogeneous active α-satellite arrays and surrounding pericentromeric sequences. These regions are enriched for sequence divergence, segmental duplications, and signatures of structural turnover, and exhibit greater genomic and epigenetic variability than the highly homogenized core α-satellite arrays [36, 37]. Such features may render these regions particularly susceptible to HPV capture during DNA repair processes.

Future studies integrating HPV integration maps with epigenomic datasets, including ChIP-seq profiles for heterochromatin-associated histone marks (H3K9me3 and H4K20me3), centromere-specific histone CENP-A occupancy, chromatin accessibility measurements, and chromosome conformation analyses, will be required to determine whether HPV integrations preferentially occur within specific chromatin states and whether they contribute functionally to tumorigenesis. The availability of the complete T2T reference genome now provides the genomic framework necessary for such investigations, which were largely impossible using previous genome assemblies.

One limitation of this study is the absence of fully identical analytical pipelines compared with the original studies, which precludes direct one-to-one comparison of breakpoint calls. However, the primary objective of this work was not replication of earlier analyses but evaluation of the impact of reference genome completeness in regards to repeat-rich regions. Additional limitations include reduced read support within repetitive regions due to multimapping effects and the focus on simple viral–host junctions rather than higher-order concatemeric structures, which may represent an important component of HPV-driven genome remodeling. This study would also benefit from functional annotation of the integrations detected, however, hs1 reference so far has not been as well annotated as the previous references – this is most likely to change in the near future to enable researchers more in-depth analyses.

Importantly, our results demonstrate that improved detection of HPV integrations is not restricted to long-read sequencing technologies. Even short-read datasets revealed additional integration events when mapped against the complete T2T reference genome, indicating that reference genome completeness represents a critical determinant of integration discovery. This finding is supported by other studies where the use of the full reference genome led to better detection of specific regions [30, 38] and suggests that reanalysis of existing sequencing datasets may provide substantial additional biological insight without the need for new experimental data.

Looking forward, continued advances toward pangenome and sample-specific reference frameworks are likely to further refine viral integration analyses. Increased sequencing throughput, longer read lengths, and single-cell sequencing approaches may help distinguish between true biological breakpoint heterogeneity and technical mapping limitations within repetitive genomic regions.

In summary, our study demonstrates that adoption of the complete T2T human reference genome substantially expands the detectable landscape of HPV integration events. These findings highlight the importance of reference genome completeness for studies of virus–host genome interactions and suggest that integration events within repeat-rich genomic regions represent a previously underappreciated component of HPV-driven carcinogenesis. Whether these integrations contribute to tumorigenesis through local genome instability, disruption of centromeric chromatin, or long-range effects on chromatin architecture remains an important question for future studies.

## Conclusion

Taken together, our results demonstrate that the adoption of the complete telomere-to-telomere (T2T) human reference genome substantially expands the detectable landscape of HPV integration events. By reanalyzing previously published sequencing datasets, we show that a considerable fraction of viral integration breakpoints occurs within repeat-rich genomic regions that were absent from earlier reference assemblies and therefore remained undetected. These findings indicate that HPV integration landscapes have been systematically underestimated in prior studies, independent of sequencing technology.

## Materials and methods

### Study design and datasets

To evaluate how reference genome completeness affects detection and annotation of HPV integration breakpoints, we reanalyzed three previously published datasets encompassing four sequencing strategies: (i) PacBio continuous long reads (CLR) with HPV-targeted enrichment (Iden et al.), (ii) PacBio HiFi and (iii) Oxford Nanopore Technologies (ONT) long reads without HPV-targeted enrichment (Akagi et al.), and (iv) Illumina short-read sequencing generated from proximity ligation libraries from FFPE tumor material (Demers et al.). All analyses were performed by mapping reads to a hybrid reference consisting of the telomere-to-telomere human assembly (T2T; chm13v2/hs1) and the HPV16 reference genome. 

### Reference genomes and annotations

The human reference genome used throughout this study was the telomere-to-telomere assembly chm13v2/hs1. The viral reference was the HPV16 genome sequence. For analyses involving repeat-rich regions, centromeric satellite (cenSat) annotations were obtained from the UCSC Genome Browser database for hs1 (https://hgdownload.soe.ucsc.edu/gbdb/hs1/censat/censat.bb, version from 29th March 2022) [39–41]. The breakpoint files were converted to BED file format. The CSF sites were downloaded from https://webs.iiitd.edu.in/raghava/humcfs/index.html [42] and the positions from hg38 were lifted over to hs1 with UCSC LiftOver tool [43]. Common fragile site (CFS) intervals were obtained from HumCFS https://webs.iiitd.edu.in/raghava/humcfs/index.html (19th December 2025) [42] (originally defined on hg38) and lifted over to hs1 prior to overlap analyses. The overlap analysis was performed using GenomicRanges R package version 1.52.1 [44].

### Processing of short-read proximity ligation data (Demers et al.)

Raw Illumina sequencing reads were subjected to an initial quality assessment using FastQC (v0.12.1) [45]. Adapter sequences and low-quality bases were removed using Cutadapt (v4.4) [46]. Processed reads were aligned to the hybrid hs1 + HPV16 reference using BWA-MEM (v0.7.18) [47], which supports split and chimeric alignments. Alignments were coordinate-sorted using SAMtools (v1.18) [48]. PCR duplicates were marked and removed using Picard (v3.0.0) (https://broadinstitute.github.io/picard/).

Chimeric alignments indicative of viral–host junctions were extracted from the BAM files using SAMtools (v1.18) [48] combined with custom AWK-based filtering. Because the Demers et al. libraries were generated using an NlaIII restriction enzyme-based proximity ligation approach, we applied an additional artifact-filtering step to mitigate false-positive junctions arising from restriction site proximity. Specifically, we computed the distance between each split junction and the nearest NlaIII recognition motif (CATG/GTAC) (Supplementary Figure S6) and removed chimeric reads with a motif located within ± 10 bp of the split junction using a custom script (Supplementary Figure S7).

Filtered chimeric reads were clustered by genomic position using BEDtools (v2.31.1) [49]. To mirror the thresholding strategy used in the original study, for downstream analyses we retained clusters supported by at least n number of reads and *n* > 1% of the total chimeric reads per sample. This approach limits the impact of various number of integrations in the sample set. Candidate viral–host junctions were manually inspected in IGV (Integrative Genomics Viewer) [50] to confirm the exact breakpoint position.

In addition to our custom pipeline, we analyzed the same filtered short-read BAM files with SurVirus (release 2021), a repeat-aware viral integration caller designed for short-read sequencing data, using default parameters. Breakpoints identified by SurVirus [24] were compared with those detected by our approach.

### Processing of long-read data from Iden et al. and Akagi et al

Long-read datasets were quality-checked using NanoPlot (v1.42.0) and NanoComp (v1.12.0) [51] to assess yield, read length distribution, and per-read quality scores. Reads were aligned to the hybrid hs1 + HPV16 reference using Winnowmap2 (v2.03) [52], which is optimized for mapping long reads to repetitive reference sequences. Platform-specific presets were used: map-pb-clr for PacBio CLR, map-pb for PacBio HiFi, and map-ont for ONT reads. To improve alignment specificity in repetitive regions, masked repetitive k-mer weighting (Winnowmap2 -M option) was applied. Resulting alignments were sorted with SAMtools (v1.19.2) [48].

Chimeric long reads with split alignments spanning both the human and HPV16 references were extracted using SAMtools [48] and custom AWK-based filters. Reads were grouped into breakpoint clusters based on concordant host and viral mapping coordinates using BEDtools (v2.31.1) [49]. For each cluster, breakpoint coordinates and read support were verified by manual inspection in IGV [50]. Unless otherwise stated, breakpoints supported by at least five independent reads were retained.

### Breakpoint definition and comparison with original studies

For each viral–host junction, the integration breakpoint was defined as the genomic coordinate at which the read alignment transitions between the human and HPV16 references. Because breakpoint coordinates are inherently dependent on the reference assembly, direct coordinate-level comparison between hg19-based and hs1-based results may be ambiguous. To enable robust comparisons with the original studies (which mapped to hg19), we compared integration calls at the cytogenetic band level. Breakpoints mapping to the same cytoband were considered potentially representing the same integration event. This approach minimizes the impact of assembly-specific coordinate shifts while preserving biologically meaningful genomic localization. Where informative, UCSC LiftOver [43] was additionally used to evaluate coordinate correspondence across assemblies.

In downstream summaries, multiple breakpoints located within the same cytogenetic band in a given sample were treated as a single integration event, under the assumption that closely spaced breakpoints likely reflect a common underlying integration locus or local rearrangement around an insertion site.

### Overlap with satellite and common fragile sites

To identify integrations occurring within repeat-rich regions, breakpoint coordinates were intersected with hs1 cenSat annotations downloaded from UCSC [39, 41] (https://hgdownload.soe.ucsc.edu/gbdb/hs1/censat/censat.bb, version from 29th March 2022) using GenomicRanges R package [44]. Breakpoints overlapping cenSat intervals were classified by satellite type according to the cenSat annotation track.

To assess overlap with common fragile sites (CFSs), CFS intervals were downloaded from HumCFS [42] and lifted over from hg38 to hs1 using UCSC LiftOver [43]. Lifted intervals were then intersected with breakpoint coordinates using GenomicRanges R package version 1.52.1 [44]. Statistical enrichment of breakpoints within annotated regions was evaluated using proportion-based tests as described in the Results.

## Supplementary Information

Supplementary Material 1

Supplementary Material 2: Table S1 – List of breakpoints identified in each study filtered by ≥ 5 supporting reads.

## Data Availability

The datasets analyzed during the current study are available in the NCBI BioProject repository, [https://www.ncbi.nlm.nih.gov/bioproject/PRJNA640649](https:/www.ncbi.nlm.nih.gov/bioproject/PRJNA640649) (Iden *et al.*) and in the European Genome-phenome Archive, [https://ega-archive.org/datasets/EGAD50000001302](https:/ega-archive.org/datasets/EGAD50000001302), [https://ega-archive.org/datasets/EGAD50000001304](https:/ega-archive.org/datasets/EGAD50000001304) (Akagi *et al.*)The datasets analyzed during the current study are not publicly available due patient sensitive information but are available from the corresponding authors of the original studies on reasonable request (Demers *et al.*).

## Abbreviations

CFSs: Common fragile sites
ecDNA: Extrachromosomal circular DNA
FFPE: Formalin–fixed paraffin–embedded
HIVID: High–throughput viral integration detection
HNSCC: Head and neck squamous cell carcinomas
HPV: Human papillomavirus
ONT: Oxford nanopore sequencing technology
PacBio: CLR–PacBio continuous long read
PacBio: HiFi–PacBio high fidelity sequencing
T2T: Telomere–to–telomere
