# Supplementary material for: Revisiting HPV integration landscapes using telomere-to-telomere human genome reference closes the gaps in HPV integration sites search
Source: Virol J. 2026 Jul 9;23:213. doi: 10.1186/s12985-026-03241-y (PMC13579712; doi:10.1186/s12985-026-03241-y)
Supplement: Supplementary file 1 [file 12985_2026_3241_MOESM2_ESM.pdf]

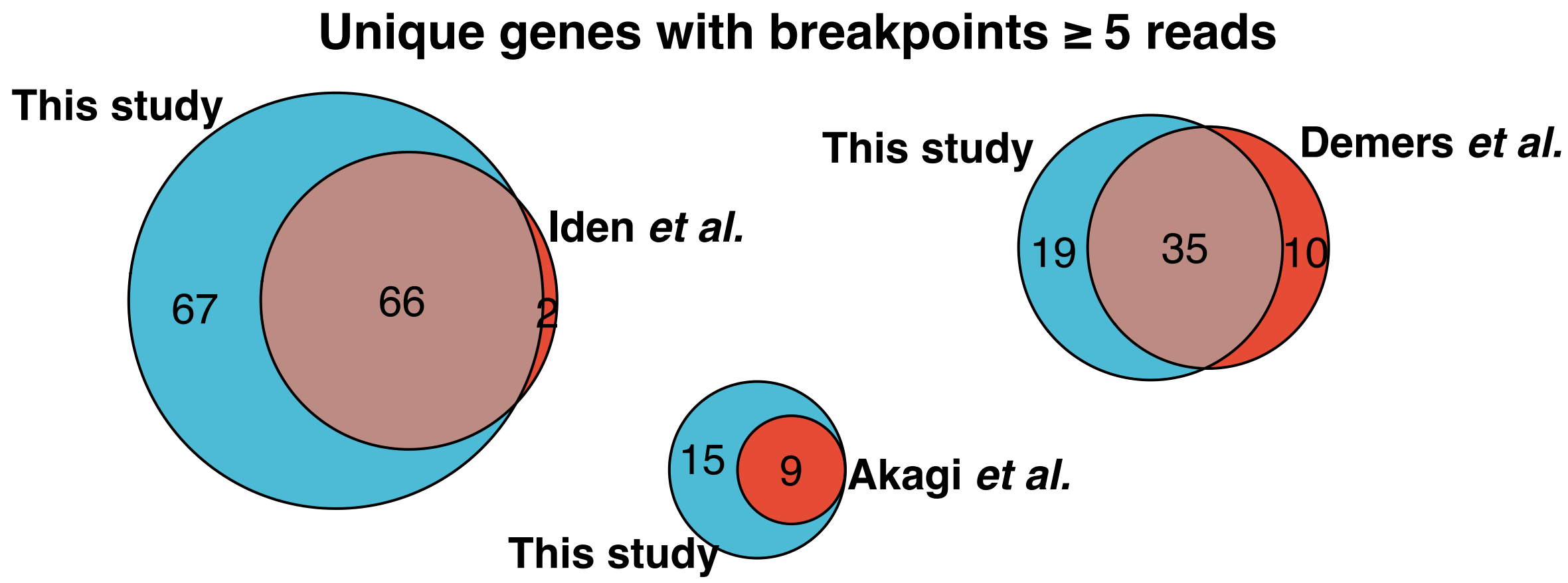

Supplementary Figure S1 – Overlap between unique genes with breakpoints (≥ 5 supporting reads) identified in this (blue) and original studies (red).

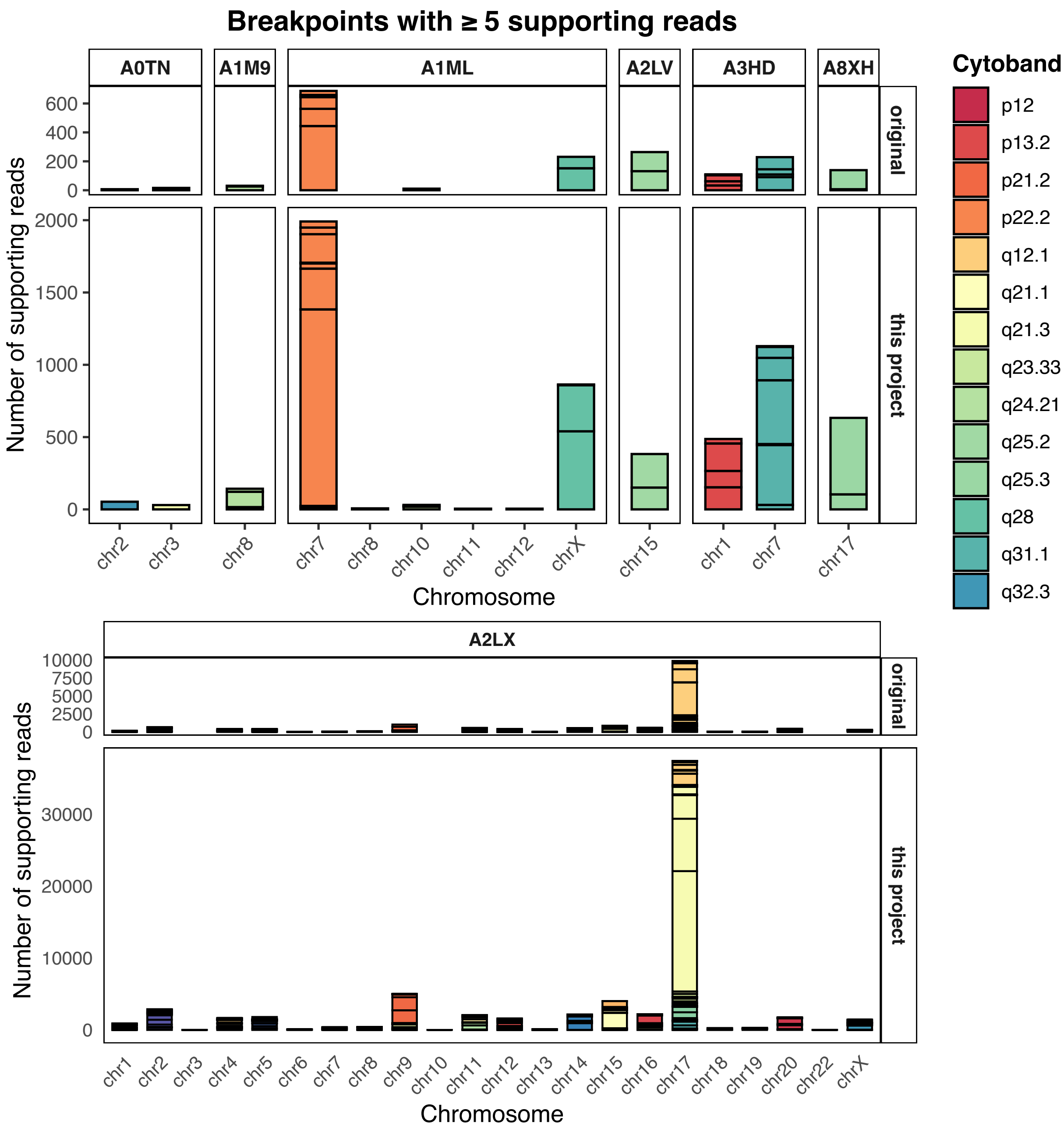

Supplementary Figure S2 – Comparison of breakpoints identified by Iden *et al.* and this study. The stacked bars show the number of breakpoints identified for each sample separated by the horizontal bars with the height of the bar indicating the number of supporting reads. If the breakpoints are on the same cytoband in both studies, they are highlighted in the same color. Cytoband information is not given for sample A2LX for visibility reasons; please refer to (Supplementary Table S1). Bars may overlap if the number of supporting reads is not clearly visible. Only breakpoints with at least five supporting reads are shown.

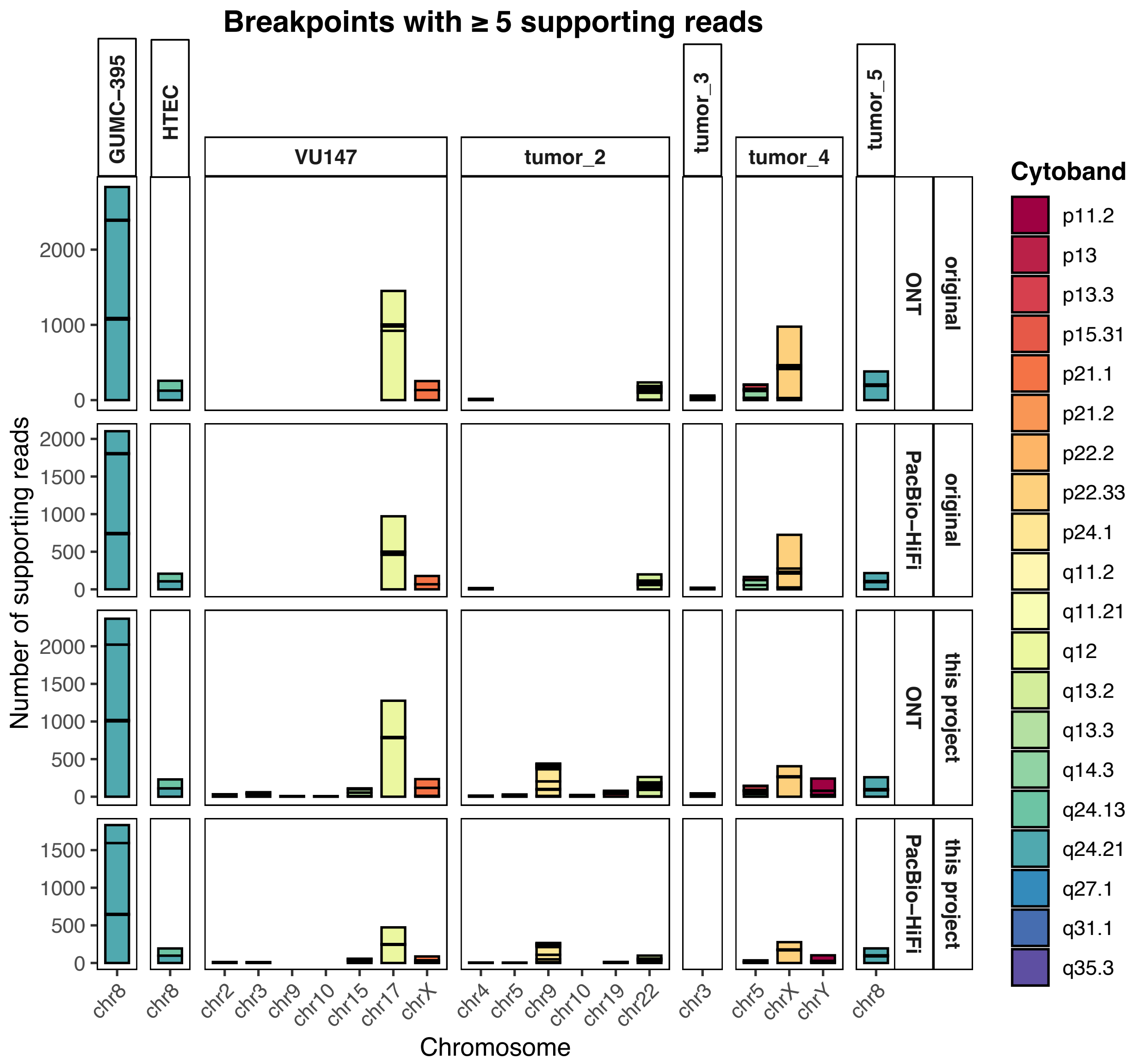

Supplementary Figure S3 – Comparison of breakpoints identified by Akagi *et al.* and this study. The stacked bars show the number of breakpoints identified for each sample separated by the horizontal bars with the height of the bar indicating the number of supporting reads. If the breakpoints are on the same cytoband in both studies, they are highlighted in the same color. The number of supporting reads per breakpoint reflects both sequencing platforms (PacBio-HiFi and ONT).

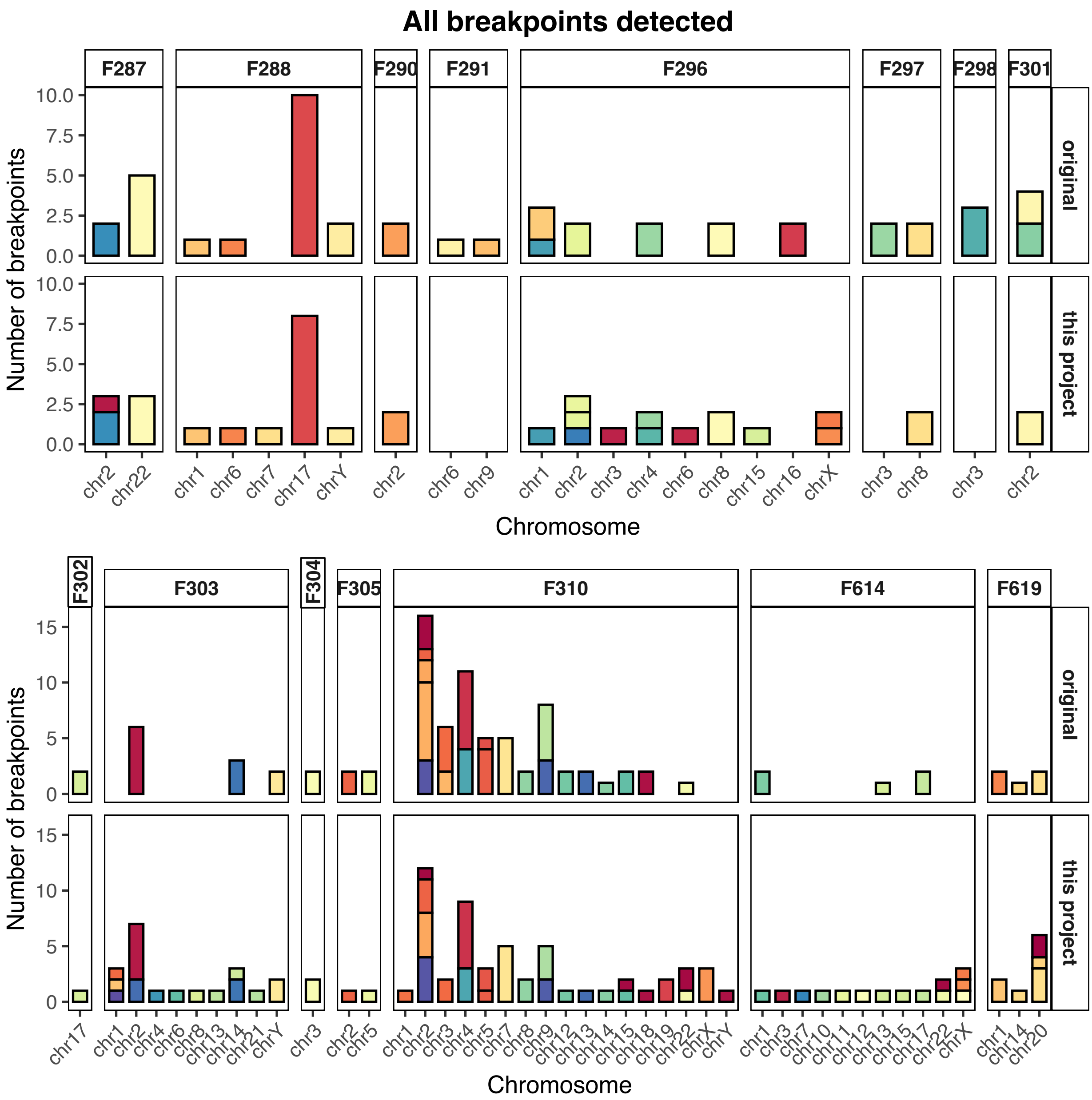

Supplementary Figure S4 – Comparison of breakpoints identified by Demers et al. and this study. Demers et al. did not provide the number of supporting reads and therefore the stacked bars show the number of breakpoints identified for each sample separated by the horizontal bars with the height of the bar indicating the number of breakpoints within each cytoband. If the breakpoints are on the same cytoband in both studies, they are highlighted in the same color.

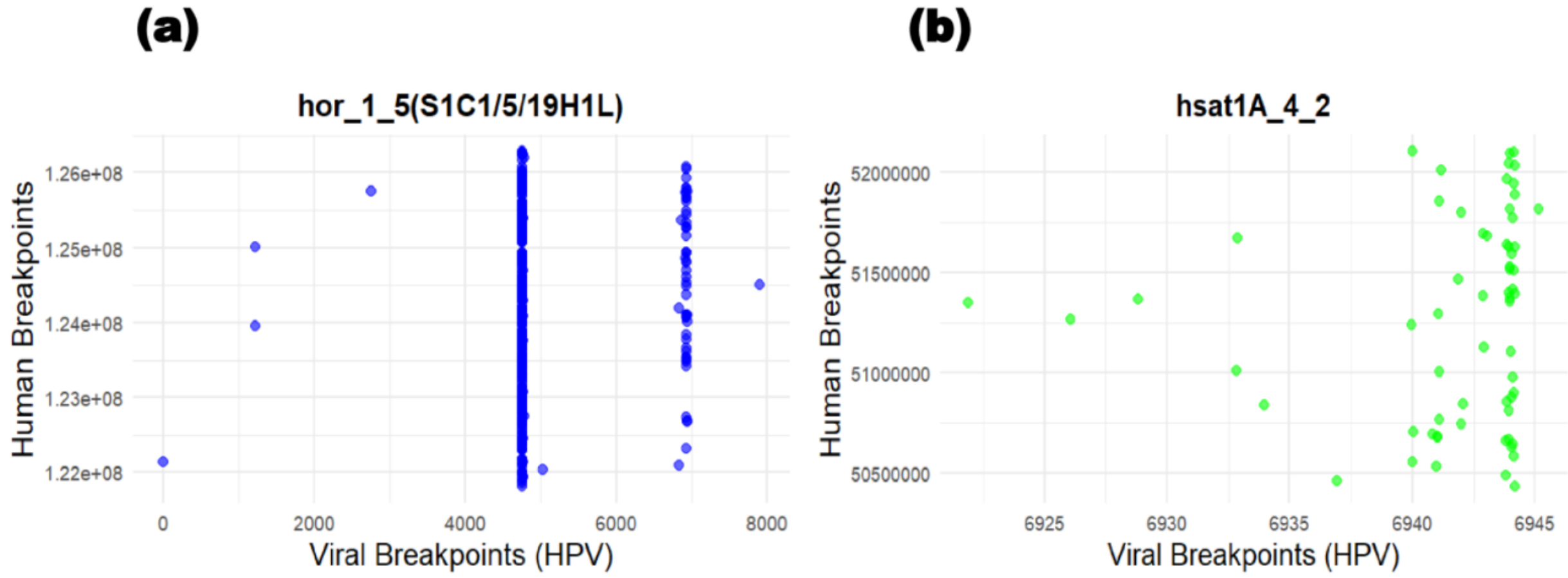

Supplementary Figure S5 – Examples of breakpoint cluster within the repeats identified in A2LX sample from Iden *et al.* study [16]: Variable human breakpoint position and consistently the same viral breakpoint position observed in the centromeric and pericentromeric repeat arrays. **A** – Multiple breakpoints identified on a major centromeric satellite repeat hor_1_5(S1C1/5/19H1L), whereas the breakpoints on the viral genome were almost exactly at the same position. This figure suggests there are two breakpoints, but the reads are scattered on the human genome, which is why the coverage of the breakpoint was usually low (< 10 reads). **B** – Another example of the breakpoint cluster on the human satellite (hsat1A_4_2) with most likely one or two breakpoints in the host genome.

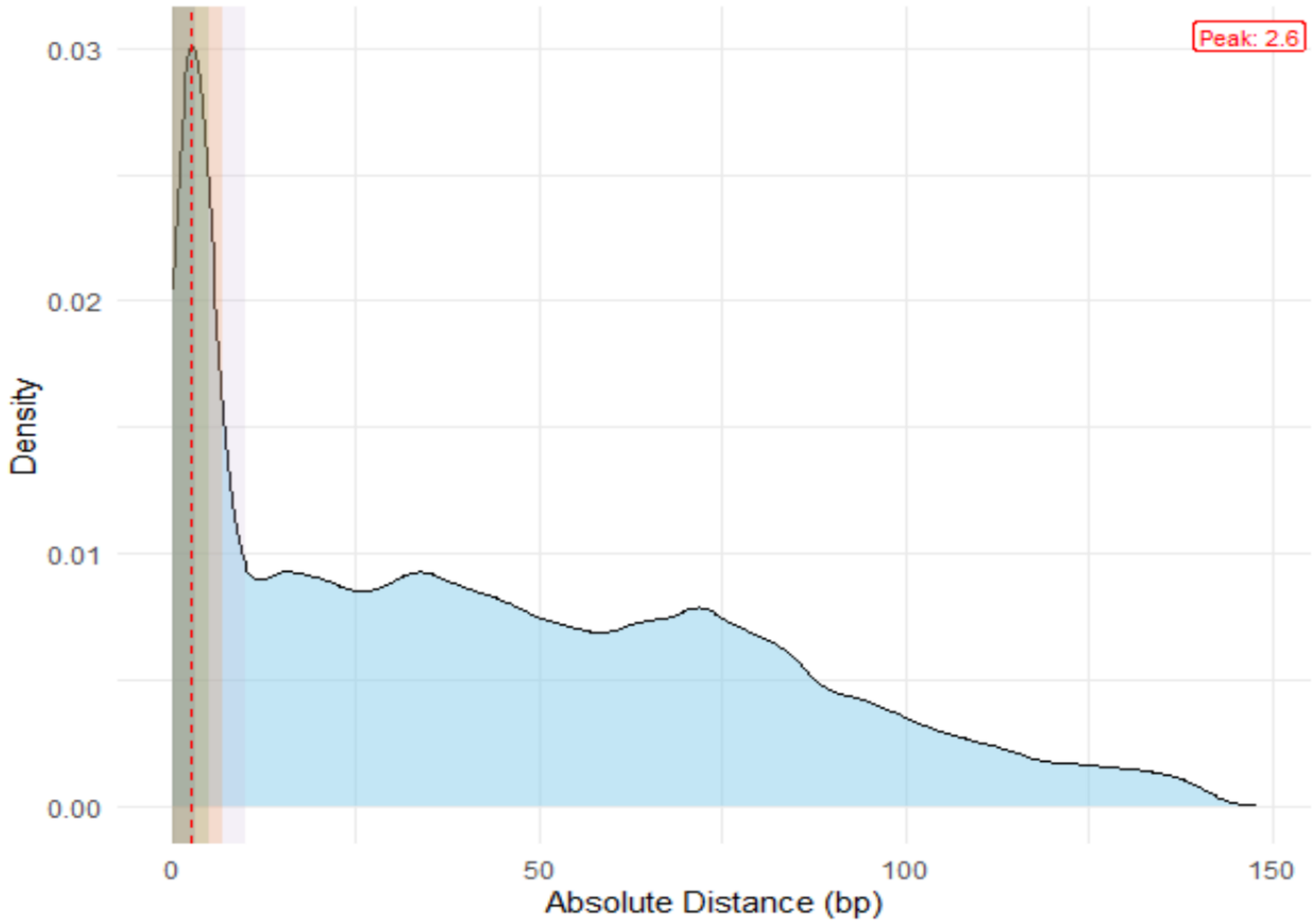

Supplementary Figure S6 – Distance of NlaIII restriction motif (CATG/GTAC) from the split junction of chimeric reads. The distance distribution is shown for all samples from Demers et al. [17]. The distance was calculated for all of the chimeric reads with the CATG/GTAC restriction motif from the split junction.

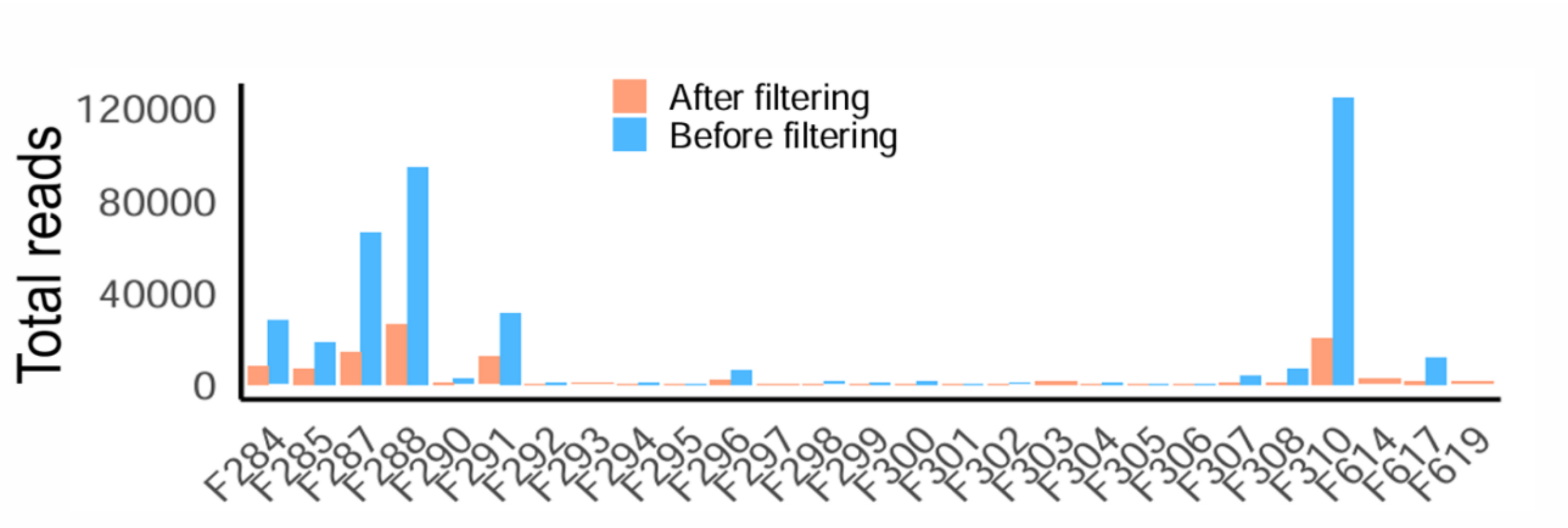

Supplementary Figure S7 – Chimeric reads from Demers et al. before and after filtering for proximate NlaIII restriction motif (CATG/GTAC). Chimeric reads that had the NlaIII cut site motif (CATG/GTAC) within 10 base pairs from the split were filtered out to remove any false positive viral-human junctions. The maximum number of chimeric reads was detected for samples F310, F288, and F288. For more than half of the samples, it was less than 1,000.
